# Endothelin receptor antagonists (ERAs) can potentially be used as therapeutic drugs to reduce hypertension caused by small molecule tyrosine kinase inhibitors (TKIs)

**DOI:** 10.3389/fphar.2024.1463520

**Published:** 2025-01-09

**Authors:** Qingjian He, Junling Lin, Chanjuan Mo, Guodong Li, Jianzhong Lu, Qiyin Sun, Lijun Cao, Haojian Gan, Quan Sun, Jiafang Yao, Shengyi Lian, WenJuan Wang

**Affiliations:** ^1^ Department of Breast and Thyroid Surgery, First Affiliated Hospital of Huzhou University, Huzhou, China; ^2^ Department of Cardiovascular Center, First Affiliated Hospital of Huzhou University, Huzhou, China; ^3^ School of Medicine, Huzhou University, Huzhou, China

**Keywords:** hypertension, tyrosine kinase inhibitors, endothelin receptor antagonists, endothelin, aprocitentan

## Abstract

The emergence of targeted anti-tumor drugs has significantly prolonged the lifespan and improved the prognosis of cancer patients. Among these drugs, vascular endothelial growth factor (VEGF) inhibitors, particularly novel small molecule tyrosine kinase inhibitors (TKIs), are extensively employed as VEGF inhibitors; however, they are also associated with a higher incidence of complications, with hypertension being the most prevalent cardiovascular toxic side effect. Currently, it is widely accepted that TKIs-induced hypertension involves multiple mechanisms including dysregulation of the endothelin (ET) axis, reduced bioavailability of nitric oxide (NO), imbalance in NO-ROS equilibrium system, vascular rarefaction, and activation of epithelial sodium calcium channels; nevertheless, excessive activation of ET system appears to be predominantly responsible for this condition. Moreover, studies have demonstrated that ET plays a pivotal role in driving TKIs-induced hypertension. Therefore, this review aims to explore the significance of ET in the pathogenesis of hypertension induced by targeted anti-tumor drugs and investigate the potential therapeutic value of endothelin antagonists in managing hypertension caused by targeted anti-tumor drugs.

## 1 The current status and research significance of hypertension caused by targeted anti-cancer drugs

With the advancement of targeted therapy for tumors, the survival period of tumor patients has been continuously extended, and cardiovascular events have gradually emerged as one of the significant causes of mortality in tumor patients. The primary anti-tumor drugs encompass vascular endothelial growth factor (VEGF) inhibitors (Shaw et al., 2024; Tavakolian et al., 2024). Particularly small molecule tyrosine kinase inhibitors (TKIs) that effectively inhibit signal cascades (Liu et al., 2023a). By targeting vascular endothelial growth factor receptor (VEGFR) to impede tumor angiogenesis, they have become a predominant treatment modality for numerous solid malignant tumors (Ciccarese et al., 2021; Du et al., 2022). Nevertheless, approximately one-fourth of patients using VEGF inhibitors will experience hypertension, while nearly every patient will exhibit significantly elevated blood pressure levels (Narayan et al., 2023; Pannucci et al., 2023). Cardiovascular toxic side effects including hypertension not only impact patient prognosis but also influence the efficacy of anti-tumor drugs. Therefore, the objective of anti-tumor treatment is to maximize its therapeutic effect while minimizing treatment-related cardiovascular events. It is imperative to explore novel specific therapeutic agents to facilitate seamless application of anti-tumor treatments and prevent associated cardiovascular events while improving patient prognosis.

## 2 The primary mechanism of TKIs in anti-tumor therapy

The vascular endothelial growth factor (VEGF) is the most potent factor in inducing vascular permeability and a specific mitogen for endothelial cells, playing a crucial role in promoting proliferation, migration, and angiogenesis of these cells (Liu et al., 2023b). VEGF exerts its biological effects through three main receptor subtypes: VEGFR 1–3. Among them, VEGFR2 serves as the primary mediator of VEGF’s actions and is closely associated with cell chemotaxis, division, and recombination (Sun et al., 2014). It plays a pivotal role in stimulating endothelial cell proliferation and migration while also regulating vascular permeability. VEGF signaling pathway inhibitors encompass monoclonal antibodies targeting VEGFA factor, VEGF trap, monoclonal antibodies against VEGF receptors, and TKIs. Among these options, TKIs competitively bind to the ATP site of tyrosine kinases, thereby impeding phosphorylation levels of tyrosine kinases. Consequently, tumor cell DNA repair is inhibited, G1 phase cell division is blocked, and angiogenesis suppression is achieved to exert anti-tumor effects. As a result of their efficacy in cancer treatment, TKIs have emerged as extensively utilized anticancer drugs with favorable therapeutic outcomes. However, it should be noted that cardiovascular toxic side effects such as hypertension are commonly associated with their use.

The classification of TKIs can be based on their main target of action, which includes inhibitors targeting epidermal growth factor receptor (EGFR), anaplastic lymphoma kinase (ALK), human epidermal growth factor receptor 2 (HER-2), VEGFR, Abelson murine leukemia viral oncogene (ABL), and breakpoint cluster region-fusion gene (BCR-ABL) (Réa and Hughes, 2022; Le et al., 2021). Among them, certain TKIs specifically target a single receptor, such as the widely used EGFR-TKIs osimertinib and erlotinib in lung cancer (Soria et al., 2018; Ramalingam et al., 2020). Apatinib is an innovative TKI that effectively inhibits VEGFR-2 among various tumor-related kinases (TRKs) and induces apoptosis of VEGFR-2, thereby effectively suppressing the proliferation of multiple tumor cells (Xie et al., 2021; Zhao et al., 2023). Currently, apatinib has demonstrated efficacy and safety in gastric cancer, lung cancer, and breast cancer; moreover, the combination therapy of apatinib with camrelizumab has been recommended as first-line treatment for advanced liver cancer (Xia et al., 2022; Mao et al., 2023). Meanwhile, some TKIs exhibit multi-target activity. The kinase inhibitor sunetinib specifically targets VEGFR, platelet-derived growth factor receptor (PDGFR), PDGFR-a, PDGFR-b, and other receptors (Wang et al., 2023). It is indicated for use in patients with gastrointestinal stromal tumors (GIST) who have experienced treatment failure or intolerance to imatinib therapy, as well as in patients with advanced renal cell carcinoma (RCC) who have shown no response to cytokine therapy (Jin et al., 2023; Plimack et al., 2023). The multi-targeted kinase inhibitor Anlotinib demonstrates simultaneous inhibition of VEGFR, PDGFR, and fibroblast growth factor receptor (FGFR), making it a viable third-line treatment option for patients with advanced non-small cell lung cancer (Shen et al., 2018; Lei et al., 2023). Additionally, this drug has gained approval for its efficacy in treating soft tissue sarcoma, small cell lung cancer, medullary thyroid carcinoma, Metastatic Cervical Cancer and differentiated thyroid carcinoma (Lv et al., 2022; Li, 2021; Wu et al., 2023; Xu et al., 2022). The first anti-tumor drug, sorafenib, exerts dual inhibition on Raf protein kinase (RAF) and VEGFR kinases. By suppressing the activity of VGFR-2, VGFR-3, and RAF-1, it effectively hampers tumor cell proliferation through direct blockade of the RAF/MEK/ERK-mediated signaling pathway (Wilhelm et al., 2008; Gentile et al., 2016; Kim et al., 2018). Moreover, its impact on VEGFR and PDGFR enables angiogenesis inhibition and disruption of nutrient supply to restrict tumor cell growth (Mangana et al., 2012; Fallahi et al., 2022). Sorafenib is widely recognized as a standard first-line treatment for advanced renal cell carcinoma (Hsieh et al., 2017; Sun et al., 2022).

### 2.1 Possible mechanisms of ET-1 in TKIs-induced hypertension

**FIGURE 1:**
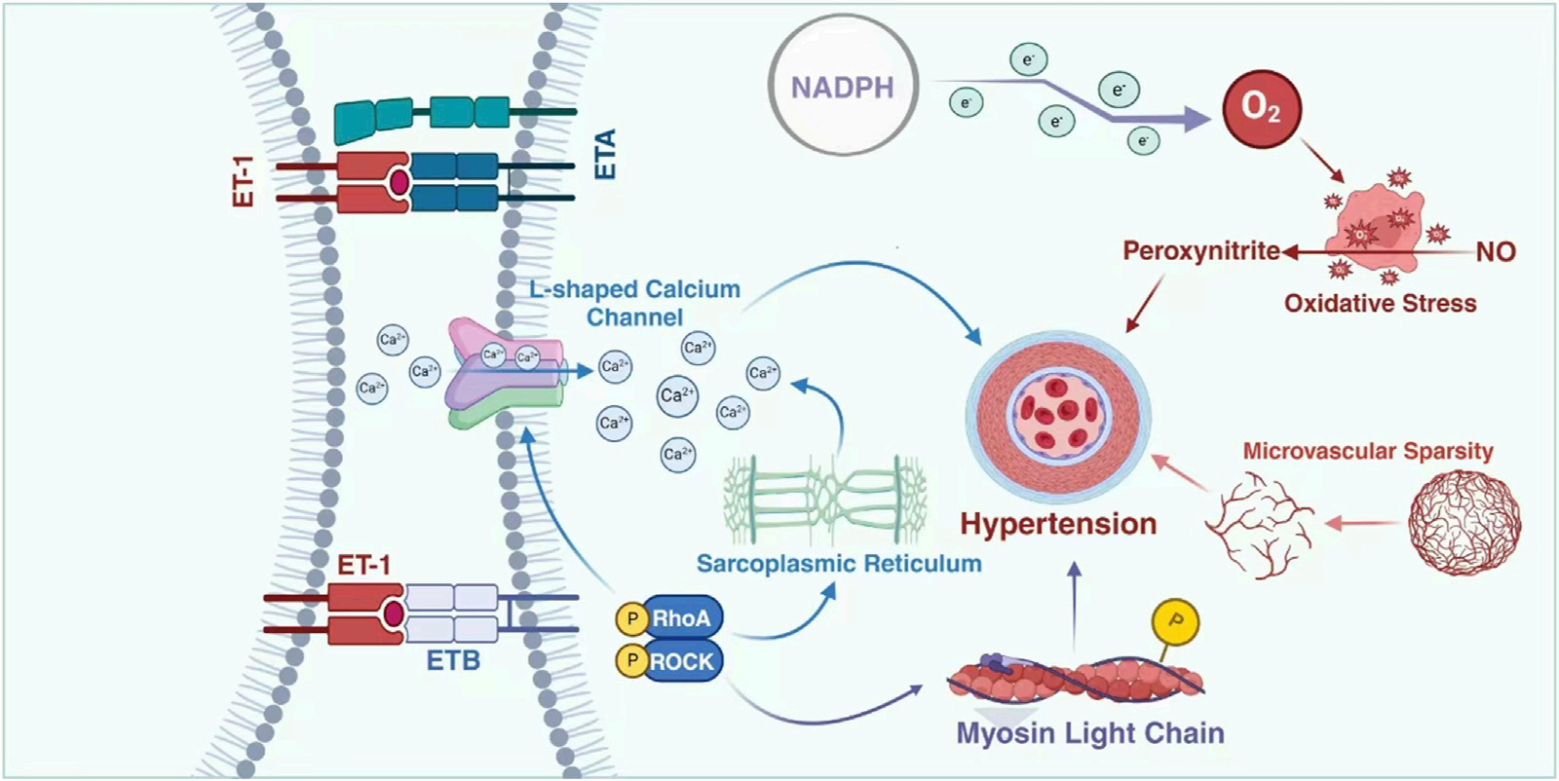
Possible mechanisms of ET-1 in TKIs-induced hypertension. In the hypertension conditions induced by TKIs, microvascular damage is highly likely to be one of the most crucial and leading factors. The substance ET can inflict damage on endothelial cells via L-type calcium channels, further intensifying this condition. This will lead to a marked reduction in the density of the microvascular network, making it increasingly rarefied. Such changes in the density and structure of the microvascular network gradually break the originally normal physiological balance and interfere with the normal functions of blood vessels, thereby gradually triggering the occurrence of hypertension. ETA: endothelin A; ETB: endothelin B; ET1: endothelin 1; ET2: endothelin 2; ET3: endothelin 3; NO: endothelial nitric; NAPDH: Nicotinamide Adenine Dinucleotide Phosphate (reduced form).

ET-1 exerts its influence on blood pressure through multiple mechanisms, rendering it an appealing therapeutic target for hypertension and other related conditions. Moreover, ET-1 plays a pivotal role in the pathogenesis of antineoplastic drug-induced hypertension, as well as being a crucial pathway involved in VEGF inhibitor-induced hypertension and renal damage (Facemire et al., 2009; Kappers et al., 2012). Studies have demonstrated that patients and animals treated with VEGF inhibitors exhibit a two-to three-fold increase in plasma levels of cleared receptors (Kappers et al., 2010). Consistent with the dose-dependent nature of elevated blood pressure, the increase in circulating ET-1 exhibited a corresponding dose-dependence during VEGF inhibition (Lankhorst et al., 2015). The mechanism by which VEGF deactivation leads to an elevation in ET-1 remains unclear. One hypothesis suggests that VEGF inactivation results in the loss of vasodilatory endothelial ETB receptors, thereby reducing ET-1 clearance and increasing its circulation (Versmissen et al., 2019). Additionally, dual ETA/B receptor antagonism or selective ETA receptor blockade has shown to prevent VEGF-inhibitor-induced hypertension, indicating that stimulation of ETA receptors by ET-1 is responsible for this condition (Carneiro et al., 2008). The utilization of sunitinib in a clinical pilot study resulted in an elevation of circulating levels of ET-1 *in vivo* (Sourdon et al., 2017). Indicating the crucial role played by endothelin in hypertension induced by antineoplastic drug therapy that should not be disregarded. (Figure 1).

## 3 The mechanisms of TKI-induced hypertension

**FIGURE 2:**
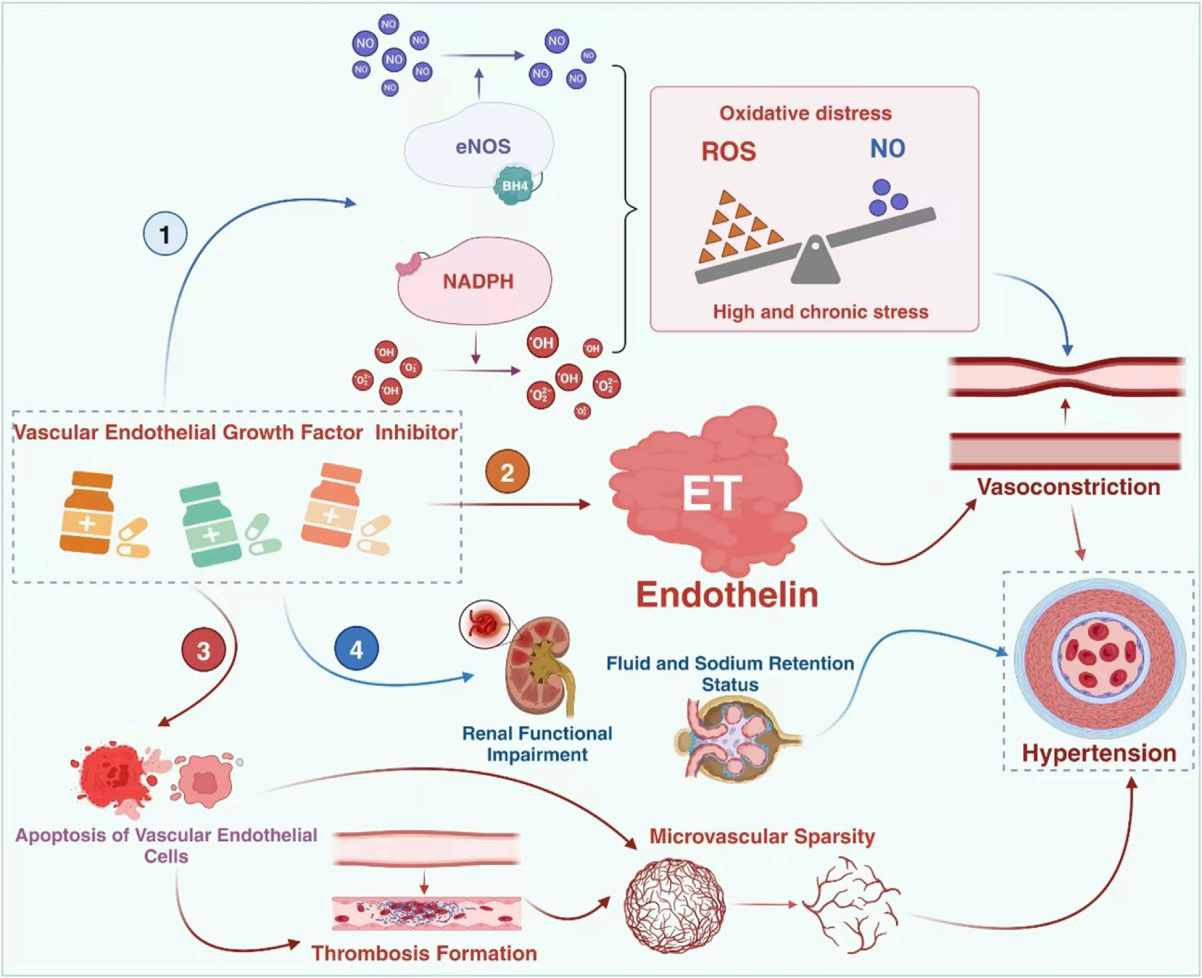
The mechanisms of TKI-induced hypertension. The mechanism of hypertension caused by vascular endothelial growth factor inhibitors may involve 4 pathways, namely the imbalance of the NO-ROS system, the impairment of the endothelin system, microvascular injury and the occurrence of renal damage. However, ET-1 might be the central link. eNOS: endothelial nitric oxide synthase; BH4: tetrahydrobiopterin.

VEGF inhibitors can increase the risk of heart failure, coronary artery disease, hypertension, and thromboembolic diseases through mechanisms such as endothelial injury, vasoconstriction and remodeling, inflammatory response, and platelet activation (Porta and Striglia, 2020; Chen et al., 2018). The potential mechanisms underlying the induction of hypertension by this class of drugs are currently believed to include (Wang et al., 2021; Wang et al., 2022a; Simonetti et al., 2009; Pérez-Gutiérrez and Ferrara, 2023; Wang et al., 2024): (i) Inhibiting nitric oxide synthase (NOS) reduces the synthesis of NO, thereby blocking its vasodilatory effects; (ii) Increased production of endothelin (ET) enhances vasoconstriction; (iii) Endothelial cell apoptosis and necrosis lead to a decrease in capillary bed density (rarefaction); (iv) Impaired renal function, increased salt sensitivity, and water-sodium retention. Among these mechanisms, the relationship between endothelin and hypertension is worth further investigation due to the varying degrees of interaction between them (Figure 2).

## 4 The upregulation of ET-1 may contribute to the development of hypertension induced by TKIs

ET-1, NO, and vascular pseudohaemophilic factor (VWF) are bioactive substances that reflect the function of vascular endothelium and play a crucial role in the occurrence and progression of cardiovascular disease and essential hypertension (Taneja et al., 2019; Yu et al., 2020). NO is a pivotal factor in endothelial nitric oxide synthase (eNOS)-mediated physiological changes in smooth muscle relaxation, which is critical for angiogenesis (Chen et al., 2024). Normal endothelial cells constitutively express eNOS; however, under pathological conditions, its inhibition leads to reduced bioavailability of NO that inhibits eNOS expression (Leo et al., 2021). Meta-analyses have demonstrated that VEGF inhibitors can increase ET-1 levels while decreasing NO expression, ultimately leading to severe cardiovascular complications such as hypertension (Xu et al., 2021).

### 4.1 Relationship between ET-1 and vasodilation and contraction

**FIGURE 3:**
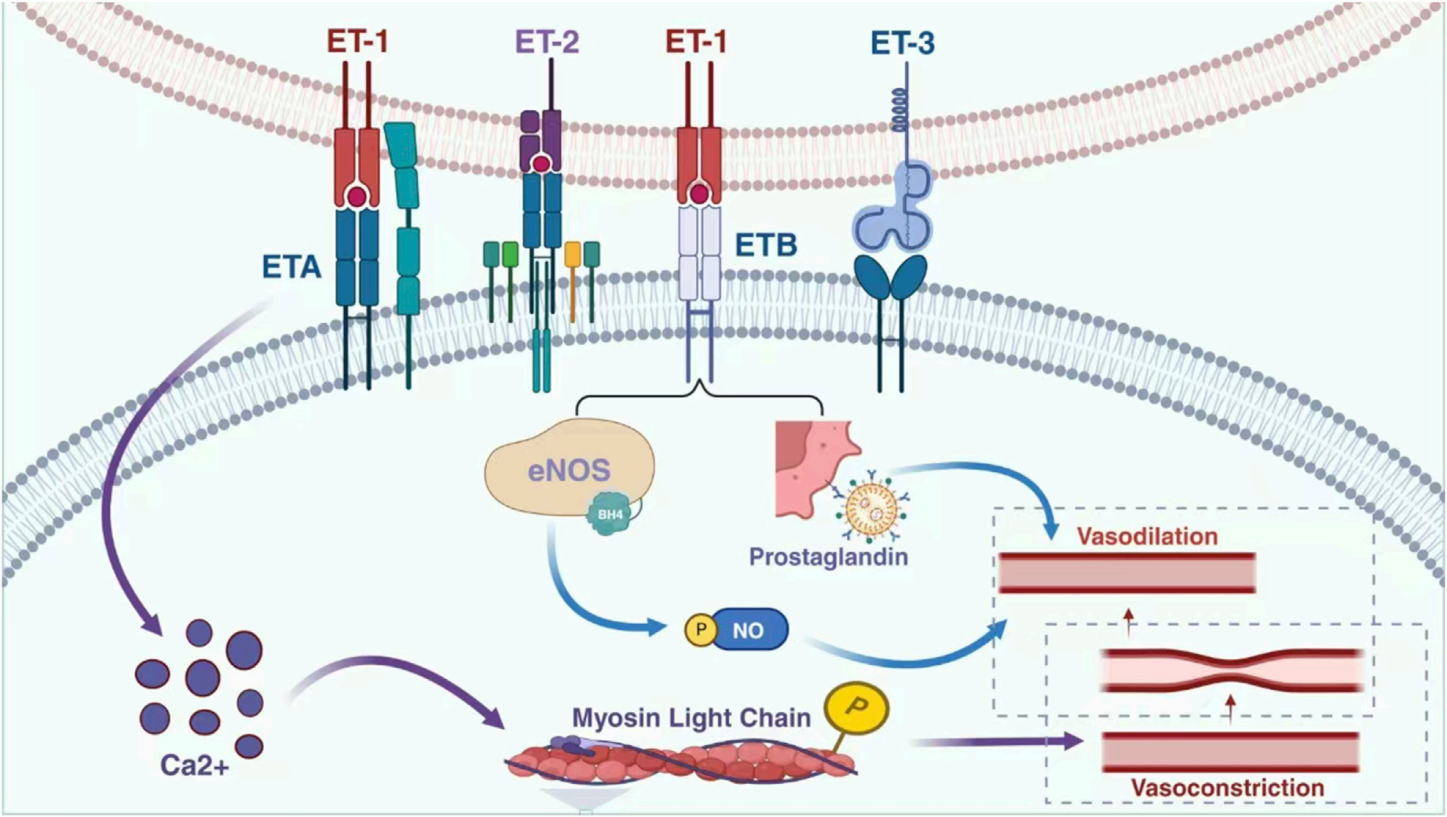
Relationship between ET-1 and vasodilation and contraction. Endothelin, a significant biomolecule, encompasses endothelin receptor A and endothelin receptor B. These receptors respectively combine with three distinct subtypes of endothelin, namely endothelin 1, endothelin 2, and endothelin 3. Through this specific interaction and combination, a physiological process is initiated that ultimately leads to vasoconstriction. This mechanism plays a crucial role in regulating vascular tone and blood flow within the complex biological systems of the body. eNOS: endothelial nitric oxide synthase; NO: endothelial nitric; ETA: endothelin A; ETB: endothelin B; ET1: endothelin 1; ET2: endothelin 2; ET3: endothelin 3.

ET was initially identified as a potent vasoconstrictive peptide for porcine aortic endothelial cells (ECs) (Ma et al., 2023). It consists of 21 amino acid residues with a hydrophobic C-terminus connected by two sets of intrastrand disulphide bonds, exhibiting vasopressor effects (Laudette et al., 2021). Three isomers of human ET exist, namely ET-1, ET-2, and ET-3 (Davenport and Maguire, 2006). Although they differ in structure and activity, all three are antihypertensive *in vivo* and exhibit strong contractile effects on vascular smooth muscle cells *in vitro*. Among these isomers, ET-1 plays a major role in regulating the cardiovascular system and vascular endothelial cells serve as the primary source of this isopeptide (Harrison et al., 2024; Finch and Conklin, 2016). In addition to ECs, various cell types including vascular smooth muscle cells (VSMCs), cardiomyocytes, fibroblasts, macrophages, epithelial cells of the lungs and kidneys as well as neurons and glial cells express ET-1 (Chen et al., 2020; Davenport et al., 2016). The degree of vasoconstrictive activity induced by these peptides follows the order: ET-2 > ET-1 > ET-3 (Inoue et al., 1989). However, ET-1 is currently recognized as the most potent vasoconstrictor known to induce constriction or relaxation in the vasculature by binding to endothelin A (ETA)/endothelin B (ETB) receptors on smooth muscle cells (SMCs) (Maguire and Davenport, 2015; Kumar et al., 2013). The interaction between ET-1 and ETA receptors on vascular smooth muscle cells leads to an increase in intracellular calcium levels, while the interaction between ETA receptors further elevates intracellular calcium levels (Ortega Mateo and de Artiñano, 1997; Hopfner et al., 1998). This rise in intracellular calcium triggers phosphorylation and activation of myosin light chains, ultimately resulting in vasoconstriction. Similarly, binding of ET-1 to ETB receptors on endothelial cells activates both eNOS and prostaglandin pathways, leading to vasodilation of the vasculature (Wolf et al., 2014; Leite et al., 2013; Neves et al., 2019) (Figure 3).

### 4.2 Key factors involved in the ET-1 induced hypertension

#### 4.2.1 NO-ROS imbalance

The exploration of the mechanism underlying hypertension induced by antineoplastic drugs has suggested a correlation with ROS oxidative stress triggered by ET-1, which enhances NADPH oxidase activity and consequently elevates ROS production. This, in turn, may contribute to hypertension by further diminishing available NO, thereby challenging the disassociation of ET-1from the NO-ROS equilibrium (Camarda et al., 2022; Wilcox et al., 2019; Muhammad et al., 2020).

Oxidative stress resulting from excessive production of ROS is a crucial mechanism underlying endothelial cell death (Zheng et al., 2022; Fan et al., 2024; Lin et al., 2022). ROS, such as superoxide and hydrogen peroxide, have been identified as molecules involved in angiogenesis. While excessive ROS levels induce senescence and apoptosis in endothelial cells and stem/progenitor cells, low concentrations of ROS play a pivotal role in redox signaling pathways that mediate angiogenesis (Ushio-Fukai and Alexander, 2004; Yingze et al., 2022). During antiangiogenic therapy, oxidative stress may contribute to the development of hypertension by oxidizing NO to peroxynitrite, thereby reducing NO-mediated vasodilator tone (Sedeek et al., 2003). The literature has extensively investigated whether the elevation of MAP induced by sunitinib is accompanied by an increase in oxidative stress markers and if antioxidants can prevent or reverse this potential rise in MAP (Kappers et al., 2011; Lankhorst et al., 2014). Through studying the role of oxidative stress in hypertension, it has been determined that upregulation of ROS production is more likely to be a consequence of VEGF inhibitor-induced hypertension (Versmissen et al., 2019). The reduced availability of NO plays a crucial role in the development of hypertension induced by VEGF inhibitors, while it has been demonstrated that ET-1 enhances NADPH oxidase activity, leading to increased production of ROS and potentially contributing to hypertension by further diminishing the levels of available NO (Kappers et al., 2011; Sarkar et al., 2019). ETA receptors are implicated in blood pressure regulation as well as chemotaxis, release of inflammatory mediators, generation of ROS, and neointimal formation associated with vascular remodeling processes (Eid, 2024; Sobrano Fais et al., 2023).

#### 4.2.2 Microvascular damage

Microvessel thinning (reduced microvessel density), leading to impaired microcirculation and increased vascular resistance. This is one of the mechanisms of VEGF inhibitor-induced hypertension (Touyz and Herrmann, 2018). Microvessel thinning was initially speculated to be possibly functional, and with intense vasoconstriction, and later structural, then the relationship between endothelin and microvessel thinning had to be revisited. There are experimental and clinical data suggesting that ET-1 is responsible for maintaining arterial stiffness (Amiri et al., 2004). Increased ET-1 activity may lead to atherosclerosis and atherosclerotic arterial stiffness (Sedeek et al., 2003; Kostov, 2021; Gu et al., 2015). These pathological processes significantly reduce the elastic properties of central conduit arteries, leading to the manifestation of isolated systolic HTN. Increases in systolic and central pulse pressure may lead to eutrophication or hypertrophic remodelling of small arteries. In particular, hypertrophic remodelling of resistant arteries is a hallmark of ET-1 involvement in the hypertensive process.

VEGF inhibition resulted in a decrease in the number of perfused capillary networks, leading to an elevation in total peripheral resistance (TPR) and blood pressure. Capillary atrophy is attributed to endothelial cell apoptosis and chronic remodeling of the microvasculature observed during VEGF occlusion (Iwagawa et al., 2023). In one study, sunitinib-treated patients were examined for capillary density using microscopy (Gu et al., 2015). The researchers discovered that a reduction in microvascular network density during tki treatment was correlated with an increase in blood pressure. The reduced density of microvessels also results in obstructed microcirculation and increased vascular resistance. In a study involving patients with colorectal cancer, bevacizumab was administered and after 6 months of treatment, the patients’ blood vessels were evaluated. Moderate vasodilution was observed, suggesting that vasodilution may be one of the mechanisms by which VEGFI induces high blood pressure (Mourad et al., 2008). Furthermore, it is likely that vascular thinning is functional rather than structural (Chuang et al., 2018). Additionally, endothelial cell dysfunction may contribute to thrombosis, leading to further reduction in vascular perfusion, increased apoptosis, and loss of microvasculature.

#### 4.2.3 Activation of related signalling pathways

Previously, our team’s study has demonstrated that apatinib can induce hypertension in WKY rat models through the activation of the RhoA/ROCK pathway, specifically via Rho protein kinase (ROCK) (Li et al., 2022). Furthermore, we simulated the tumor microenvironment for *in vivo* experiments and discovered that the RhoA/ROCK signaling pathway is also implicated in apatinib-induced hypertension and vascular remodeling mechanisms in mice with gastric cancer (Wang et al., 2022b; Wang et al., 2022c).

In recent years, it has been proposed that ET-1 acts as an upstream effector, stimulating the phosphorylation of myosin light chain (MLC) in vascular smooth muscle through the RhoA/ROCK pathway (Hwang et al., 2024). Simultaneously, it enhances oxidative stress in vascular endothelial cells via this pathway, leading to increased peroxide production and subsequent reduction in NO production (Choraghe et al., 2020). These effects ultimately contribute to vasoconstriction and the development of hypertension. Consequently, high blood pressure ensues. Prolonged (3 months) endothelium-specific overexpression of ET-1 not only results in persistent elevation of blood pressure, but also induces endothelial and renal damage (Coelho et al., 2018; Zou et al., 2019). Furthermore, the vasodilatory effects of NO are mediated through the RhoA/ROCK pathway and NO can exert its vasodilatory effect via the RhoA/ROCK pathway (Kai et al., 2019). Moreover, several studies have confirmed that the RhoA/ROCK pathway plays a crucial role in the development of salt-sensitive hypertension and hypertension-induced cardiac hypertrophy (Cao et al., 2016; Kobayashi et al., 2009). The vasoconstrictive effect of ET-1 can be potentiated by the activation of the RhoA/ROCK pathway, which is implicated in reducing vasodilator function by enhancing oxidative stress, promoting peroxide production, and diminishing endothelial NO production (Zhuang et al., 2018; Tsai et al., 2017). Notably, ET-1 plays a pivotal role in mediating TKIs-induced hypertension and serves as a key driver for this condition.

#### 4.2.4 Regulation of calcium ions

The study demonstrated that ET-1-mediated vasoconstriction is associated with calcium ions, and experimental evidence also confirmed the coupling of receptor-specific calcium signaling cascades to endothelin ETA and ETB receptors in drug-resistant arteries (Abdel-Samad et al., 2016). Endothelin ETA primarily mediates ET-1-induced vasoconstriction, with a minor contribution from VSM endothelin ETB receptors. This process involves mobilization of Ca^2+^ from intracellular stores, activation of nonselective cationic TRPC3 channels, entry of extracellular Ca^2+ t^hrough dihydropyridine-sensitive L-type channels, and mechanisms enhancing Ca^2+^ sensitivity (Peppiatt-Wildman et al., 2007; Adebiyi et al., 2012). Additionally, protein kinase C (pKC) plays a crucial role in regulating Ca^2+^ handling by augmenting voltage-dependent Ca^2+^ influx (Ziemba and Falke, 2018). Furthermore, regulation of Ca^2+^ handling serves a dual purpose through a feedback loop that inhibits release of intracellular SR stores’ stored Ca^2+^. ROCK not only regulates intracellular Ca^2+^ mobilization and entry but also acts as a major determinant for increased myofilament sensitivity to vasoconstriction induced by activation of ET receptors in drug-resistant arteries (Rattan, 2017; Zhao et al., 2021). Enhanced ROCK activity contributes to increased Ca^2+^ sensitization, vasoconstriction, and vascular remodeling in hypertension. Additionally, PKC-mediated increases in L-type Ca^2+^ entry are associated with abnormal vasoconstriction in insulin-resistant states (Zhang et al., 2003). Therefore, these kinases represent potential pharmacological targets for vascular diseases that involve impaired ET pathways.

## 5 Traditional antihypertensive treatment regimens

The position paper on tumor treatment and its impact on cancer and cardiovascular toxicity suggests that medication recommendations for high blood pressure can enhance the prognosis of patients in the long term, particularly through the use of angiotensin converting enzyme inhibitors (ACEIs), angiotensin receptor blockers (ARBs), β-receptor blockers (Zamorano et al., 2016). Similarly, both cardio-oncology guidelines in the United States and Europe continue to advocate ACEIs/ARBs as the primary choice for hypertension management (Alexandre et al., 2020). However, non-dihydropyridine calcium channel blockers (CCB) are generally not recommended due to their potential interaction with VEGF-targeted therapy. Diuretics are used carefully, because diarrhea or using diuretics cause electrolyte can increase the risk of QT extension. If cause liver toxicity, VEGF inhibitors should be careful to use the CCB. When TKIs cause bradycardia should be careful to use beta-blockers drugs. The different classes of antihypertensive medications commonly interact with antitumor drugs see in Table 1.

**TABLE 1:** The commonly employed antihypertensive agents exhibit interactions with anti-tumor drugs.

Types of antihypertensive drugs	Common drug interactions with anti-tumor drugs
Beta blocker	• TKIs (such as imatinib and gefitinib) may increase metoprolol blood pressure concentration
	• TKIs (such as Seretinib and Clozotinib) combined with all beta blockers may further exacerbate bradycardia
	• The combination of beta blockers and all TKIs that prolong the QT interval may lead to worsening of QT interval extension
	• Carvedilol interacts with afatinib and Venetok and should be avoided from use
Calcium channel blockers	• Avoid using CYP3A inhibitors such as diltiazem, verapamil, and felodipine, as they cause elevated plasma levels in most TKIs
	• Verapamil reduces the excretion of doxorubicin, paclitaxel, and irinotecan, leading to increased cardiac toxicity of these drugs
	• Felodipine exacerbates sorafenib induced hypertension through CYP3A4
	• Avoid using amlodipine with edranib, as the latter increases amlodipine levels
	• When TKIs cause liver toxicity, avoid using amlodipine
ACEIs	• Combined use with mTOR inhibitors increases the risk of vascular edema
Thiazide diuretics	• May exacerbate bone marrow suppression caused by cyclophosphamide
Loop diuretic	• May increase cisplatin related nephrotoxicity and ototoxicity
	• May lead to electrolyte imbalance, resulting in prolonged and worsened QT intervals related to TKIs
	• Furosemide increases the toxicity of methotrexate
Potassium preserving diuretics	• The combination of mineralocorticoid receptor antagonists (such as spironolactone and epinephrine) and some

## 6 Potential therapeutic targets of TKIs for enhancing blood pressure through modulation of the endothelin system

The pathophysiological effects of ET-1 are primarily mediated through ETA isoforms. In preclinical and acute experimental studies, highly selective peptide antagonists targeting ETA (including BQ123 and TAK-044) as well as ETB (BQ788), along with three nonpeptide antagonists (bosentan, macitentan, and ambrisentan), which either exhibit hybrid ETA/ETB antagonist properties or demonstrate ETA selectivity, have been clinically approved for use primarily in the treatment of pulmonary hypertension (Maguire and Davenport, 2015; Bonvallet et al., 1993; Watanabe et al., 1995; Mazzotta et al., 2023). With further research, endothelin receptor antagonists may also find application in the management of refractory hypertension. A variety of ETRAs have been developed and are categorized into three groups based on their functions: selective ETAR antagonists (ETARA), such as darusentan and ambrisentan (Enseleit et al., 2008; Croxtall and Keam, 2008). Selective ETBR antagonists (ETBRA) like bosentan and non-selective ETRAs including macitentan (Hosseinbalam et al., 2023; Grünig et al., 2024). Additionally, there are non-selective ERA aprocitentan (Dhillon, 2024).

### 6.1 The progression of antihypertensive effects exerted by endothelin and its antagonists

Endothelin antagonists can be utilized for the treatment of essential and refractory hypertension, with bosentan being the first ETRA employed in clinical trials for hypertension management. The findings from this study demonstrated that bosentan alone exhibited a statistically significant reduction in blood pressure after 4 weeks among patients with essential hypertension, comparable to the antihypertensive effect of enalapril (Karam et al., 1996). Another study revealed that darusentan could potentially exert an evident antihypertensive effect in treating essential hypertension (Black et al., 2007). However, its efficacy was not superior to ACE inhibitors. Moreover, serious hepatic damage, pulmonary arterial dilatation, and other adverse effects associated with darusentan use, thereby limiting further exploration into monotherapy for essential hypertension treatment. In terms of the antihypertensive effect of different drugs in ETRA, it has been newly reported that in animal experiments, the new drug macitentan has a stronger antihypertensive effect than bosentan, and it can also be clinically studied (Iglarz et al., 2014). Aprocitentan helps to dilate blood vessels and lower blood pressure by antagonising ETA receptors. Aprocitentan is commonly used in the treatment of refractory hypertension, patients with hypertension that has failed to respond to other medications, either as monotherapy or in combination with other antihypertensive medications (Georgianos and Agarwal, 2023). Clinical trials have shown that Aprocitentan provides clinically meaningful reductions in systolic blood pressure (SBP) and diastolic blood pressure (DBP) in the treatment of patients with refractory hypertension, that such reductions in blood pressure can be sustained for up to 48 weeks, and that its adverse effects can be controlled and the adverse effects are controllable (Schlaich et al., 2022).

### 6.2 Potential targets for endothelin receptor antagonists in hypertension caused by antitumor therapy

Endothelin is not only associated with cancer, but also implicated in the adverse effects induced by antineoplastic drugs, particularly hypertension. Studies have demonstrated a significant elevation of ET-1 levels during the treatment of patients across various types of cancers (Aliabadi et al., 2022; Tapia and Niechi, 2019; Rosanò and Bagnato, 2016). Targeting the ET axis and inhibiting it through specific, selective, and dual-competitive ET receptor antagonists represents an appealing approach for cancer therapy (Tocci et al., 2023). Currently, ETA and/or ETB antagonists are undergoing clinical trials to evaluate their efficacy in diverse indications such as cardiovascular disease and cancer.

It has been demonstrated that the targeting mechanism of anti-tumour drugs is intricately associated with ET-1, while cancer metastasis relies on neovasculogenesis. Tumour cells secrete factors that stimulate angiogenic pathways to facilitate rapid growth and formation of new microvessels in a state of uncontrolled cell proliferation (Farhan et al., 2020). One approach employed by targeted anti-tumour drugs involves activating the endothelin system to induce vasoconstriction and thinning.

Activation of the ET system is a crucial factor contributing to the adverse effects caused by VEGF inhibitors, thereby favoring the utilization of ET receptor antagonists as a means to mitigate these undesired side effects. Selective ETA receptor antagonists exhibit promising potential in this regard. In a study investigating the cardioprotective effects of macitentan on animals treated with sunitinib, it was determined that concurrent administration of macitentan effectively prevented sunitinib-induced hypertension while also improving ejection fraction and reducing cardiac fibrosis (Sourdon et al., 2021).

However, ET receptor antagonists are currently not approved for the treatment of systemic hypertension or renal injury, and there is also a potential risk of adverse effects associated with selective ETA receptor blockade, particularly edema (Wykoff et al., 2022). Additionally, during VEGF inactivation, ETB receptors may undergo a phenotypic switch from vasodilator to vasoconstrictor, necessitating the use of dual ET receptor antagonists (Koyama et al., 2014). Another potentially superior approach could be targeting downstream ET-1 signaling to prevent VEGF inhibitor-induced hypertension and renal injury or interfering with ET-1 upregulation.

Endothelin receptor antagonists possess potential therapeutic value not only in the management of hypertension caused by targeted antineoplastic agents, but also in directly targeting cancer itself. Macitentan can disrupt the β-arr1 signaling network by obstructing the ET-1 receptor, thereby impeding ET-1 signaling and enhancing cancer cell response to platinum-based chemotherapy (Tocci et al., 2021). Furthermore, it downregulates angiogenic and metastatic effects across various types of cancer. In a porcine model of sunitinib-induced hypertension discovered that treatment with tizosentan (a non-selective endothelin receptor antagonist) completely mitigated sunitinib-induced elevation in blood pressure effects (Kappers et al., 2012). Using a selective endothelin receptor antagonist, further elucidated that sunitinib-induced hypertension and proteinuria are mediated via ETA receptors rather than ETB receptors. This finding aligns with known ETA-mediated effects on smooth muscle cell contraction. Aprocitentan is an endogenous antagonist of the angiotensin ETA receptor, which plays a crucial role in regulating vascular tone and water-salt homeostasis (Danaietash et al., 2022). In the field of oncology and the management of tumor-induced hypertension, Aprocitentan emerges as a promising therapeutic target due to its novel mechanism, remarkable efficacy, and excellent tolerability.

## 7 The summary and future prospects

The cardiovascular toxicity and other side effects caused by VEGF inhibitors in anti-tumor therapy have become one of the main reasons limiting the anti-tumor therapy process with VEGF inhibitors. Traditional anti-hypertensive drugs are not effective for treating such hypertension, and there is a close relationship between traditional anti-hypertensive drugs and the occurrence and development of some tumors. Therefore, it is of great importance and urgent need to explore the relevant mechanisms of cardiovascular complications caused by VEGF inhibitors in anti-tumor therapy for clinical smooth application of VEGF inhibitors.

Vasoactive molecules, such as VEGF and ET, exhibit cytokine-like activity and regulate endothelial cell growth, migration, and inflammation. Several endothelial mediators and their receptors are targeted by currently approved angiogenesis inhibitors, including monoclonal antibodies against VEGF or inhibitors of vascular receptor protein kinases and signaling pathways. Pharmacological intervention that disrupts the protective function of endothelial cells can lead to similar adverse effects. Clinically, hypertension is the most common side effect associated with inhibition of the VEGF signaling pathway. Hypertension also poses a significant risk for cancer patients, as it increases mortality and morbidity related to cardiovascular disease. When hypertension reaches a certain level, cancer patients may need to discontinue antineoplastic drugs in order to prevent further elevation of blood pressure. Therefore, hypertension is not only an adverse reaction to the use of antineoplastic drugs in cancer patients but also a significant risk factor for increased mortality in this population. The management of oncological hypertension is a critical aspect that requires attention. Commonly used antihypertensive medications include diuretics, β-blockers, calcium antagonists, angiotensin-converting enzyme inhibitors, and angiotensin II receptor antagonists. However, there is a need to explore new drugs for refractory hypertension associated with cancer treatment. One promising option worth investigating is endothelin antagonists. Endothelin plays a crucial role in the pathophysiology of hypertensive complications related to cancer therapy, and inhibiting its axis can effectively reduce blood pressure levels. This research direction represents our current focus and exploration.

## Abbreviations

eNOS, Endothelial Nitric Oxide Synthase; ET-1, Endothelin 1; ETA, Endothelin A; ETB, Endothelin B; NO, Nitric Oxid; ET3, Endothelin 3; VEGF, Vascular Endothelial Growth Factor; VEGFR, Vascular Endothelial Growth Factor Receptor; TKI, Tyrosine Kinase Inhibitors; BH4, Tetrahydrobiopterin; FOXO3a, Forkhead Box O3a; AXL, AXL Receptor Tyrosine Kinase; c-MET, Hepatocyte Growth Factor Receptor; NAPDH, Nicotinamide Adenine Dinucleotide Phosphate (reduced form).
